# The chromatin code of fungal secondary metabolite gene clusters

**DOI:** 10.1007/s00253-012-4208-8

**Published:** 2012-07-20

**Authors:** Agnieszka Gacek, Joseph Strauss

**Affiliations:** 1 Fungal Genetics and Genomics Unit, Department of Applied Genetics and Cell Biology, University of Natural Resources and Life Science, University and Research Center—Campus Tulln, 3430 Tulln/Donau, Austria; 2 Health and Environment Department, Austrian Institute of Technology, University and Research Center—Campus Tulln, 3430 Tulln/Donau, Austria

**Keywords:** Secondary metabolism, Chromatin, *Aspergillus*, Histone modifications

## Abstract

Secondary metabolite biosynthesis genes in fungi are usually physically linked and organized in large gene clusters. The physical linkage of genes involved in the same biosynthetic pathway minimizes the amount of regulatory steps necessary to regulate the biosynthetic machinery and thereby contributes to physiological economization. Regulation by chromatin accessibility is a proficient molecular mechanism to synchronize transcriptional activity of large genomic regions. Chromatin regulation largely depends on DNA and histone modifications and the histone code hypothesis proposes that a certain combination of modifications, such as acetylation, methylation or phosphorylation, is needed to perform a specific task. A number of reports from several laboratories recently demonstrated that fungal secondary metabolite (SM) biosynthesis clusters are controlled by chromatin-based mechanisms and histone acetyltransferases, deacetylases, methyltransferases, and proteins involved in heterochromatin formation were found to be involved. This led to the proposal that establishment of repressive chromatin domains over fungal SM clusters under primary metabolic conditions is a conserved mechanism that prevents SM production during the active growth phase. Consequently, transcriptional activation of SM clusters requires reprogramming of the chromatin landscape and replacement of repressive histone marks by activating marks. This review summarizes recent advances in our understanding of chromatin-based SM cluster regulation and highlights some of the open questions that remain to be answered before we can draw a more comprehensive picture.

## Introduction

Recent editions of natural products databases, such as “Antibase” contain structures and chemical characteristics of almost 39,000 different microbial “secondary metabolites” (SMs) (Laatsch 2011). Such impressive variety of small organic molecules is produced by fungi and bacteria usually only under special growth conditions termed “secondary metabolism”. This physiological state follows “primary metabolism” which is essential for growth, normal development, and reproduction. In contrast, secondary metabolism is not immediately essential for the organism, but, through the production of specific metabolites, may influence the competitiveness in natural environments and thus the long-term survival and fecundity of the species. Typical examples of secondary metabolites are pigments, which absorb damaging ultraviolet radiation and thus protect the organism against DNA damage and oxidative stress (Brakhage and Liebmann 2005; Ha Huy and Luckner 1979). Other typical fungal SMs are bioactive substances like antibiotics, which restrain microbial competitors (Baquero et al. 2011, Brakhage 1998; Meloni and Schito 1991) and mycotoxins, which have significant impacts on human and animal health (Bennett and Klich 2003; Newberne 1974). Mycotoxins such as trichothecenes and aflatoxins produced by *Fusarium* and *Aspergillus* species, respectively, occur in contaminated grain, food, and feed products and act as carcinogens and mutagens (Gourama and Lloyd 1995; Trail 2009). Fungal toxins can also suppress the defense systems of plants infected by phytopathogenic fungi (Gardiner et al. 2010; Snijders 2004; Walter et al. 2010). Other secondary products, such as hydrophobins, improve dispersal of spores and play an important role in the infection process of pathogens (Aimanianda et al. 2009; Kershaw and Talbot 1998). Siderophores are needed to solubilize iron for cellular uptake when intracellular iron pools are limiting (Haas 2003; Schrettl and Haas 2011). Secondary metabolites with medical benefit include antibiotics, immunosuppressants, anti-hypercholesterol-, anti-osteoporotic-, and anti-tumor drugs (Brakhage 1998; Keller et al. 2005; Kennedy et al. 1999; Newman and Cragg 2007).

The synthesis of all these metabolites is tightly controlled to avoid production and thus resource consumption under conditions where they are not needed. They are produced usually when macro- or micronutrients become limiting or when environmental factors such as humidity, temperature, UV irradiation, salt, or unfavorable pH values challenge the regular physiology of the cells (Hoffmeister and Keller 2007; Yu and Keller 2005). Due to the medical and economic importance of fungal SMs, genetic and physiological studies dealing with conditions and signals of SM production have been the subject of intense research. It is noteworthy that, at least in *Aspergillus* species, SM production is linked with dark-induced sexual reproduction (Bayram et al. 2008; Calvo et al. 2002). In contrast, asexual reproduction and development of conidiospores is initiated when the fungal colony is exposed to an air interphase and light (Timberlake and Clutterbuck 1994). This way, a broad dispersal of conidia in aerosols is possible. Co-production of dark-induced sexual fruiting bodies with secondary metabolites, on the other hand, could have evolved because it ensured survival of the organism under competitive conditions where growth inside a substrate (dark and low oxygen concentration) would prevent aerosol dispersal of conidiospores (David Canovas, personal communication). However, it is not yet well established whether other fungi link SM production with the darkness induced sexual cycle and the ecological significance of this regulatory mechanism remains to be verified.

Some metabolic pathways such as the production of penicillin (PEN), sterigmatocystin (ST) and aflatoxin (AF) in *Aspergillus* species or deoxynivalenol (DON) and zearaleonon (ZON) in *Fusarium* species served as model systems to understand the genetics of SM production (Brown et al. 1996; Diez et al. 1990; Hohn et al. 1995; MacCabe et al. 1990; Yu et al. 1995). The regulation of secondary metabolite production, and the connection to light, has been summarized and discussed in a number of recent reviews (Bayram and Braus 2011; Calvo et al. 2002; Fox and Howlett 2008; Georgianna and Payne 2009; Schmidt-Heydt et al. 2009; Shwab and Keller 2008), and these basic aspects will not be discussed here unless they are directly related to chromatin-based regulation.

Despite the enormous number of known metabolites, it is estimated that they represent only a small fraction of SMs fungi are able to produce in their natural habitats. These conclusions are drawn from genome sequencing projects which revealed a much higher number of potential secondary metabolite genes compared to the actually known metabolites in each sequenced species (Chiang et al. 2011; Cuomo et al. 2007; Galagan et al. 2005). It is still unclear why most of the genes coding for polyketide synthases (PKSs) or nonribosomal peptide synthetases (NRPSs) are not expressed under the conditions in which we study them. The most probable reason is that usual laboratory growth conditions are significantly different from the conditions these organisms encounter in their natural habitats where they, to some extent, rely on SMs to protect their cells against harmful environmental conditions and for chemical warfare against competitors. However, at the moment, we cannot exclude the possibility that some of these predicted SM biosynthetic genes are not transcribed but persist in evolving fungal genomes without having a function in SM biosynthesis.

For some of the well-studied metabolites such as aflatoxin, sterigmatocystin, or Deoxynivalenol researchers have learned to mimic natural production conditions in the laboratory and are able to generate the crucial signals which trigger the activation of these biosynthetic pathways. Why other SM genes are not activated by the same signals remains enigmatic. From an ecological perspective, different metabolites might be advantageous in different habitats where a given species will encounter different growth conditions and competitors. A prominent example in this respect is regulation of aflatoxin production in *Aspergillus* species by the nitrogen source: whereas *Aspergillus nidulans* produces highest ST levels (immediate precursor of AF) on nitrate as sole N-source, *Aspergillus parasiticus* promotes high AF levels on ammonium (Feng and Leonard 1998).

Driven by the demand for new bio-pharmaceuticals a number of genome mining expeditions have been launched during the past years. Different strategies based on bioinformatic analysis, overexpression of biosynthetic and regulatory genes or mimicry of natural habitats by co-cultivation of fungi and bacteria have been successfully applied during the last years to activate silent SM genes (Brakhage and Schroeckh 2011; Cichewicz 2009; Fisch et al. 2009). For example, genome-wide expression profiles recorded from strains lacking or over-expressing LaeA, the broad-domain SM regulator in *A. nidulans* (see below) led to the identification of terrequinone A (TR) (Bok et al. 2006). Another fruitful approach used *trans*-overexpression of a putative regulator that was found by bioinformatics analysis to reside inside a predicted, but silent, SM gene cluster (Bergmann et al. 2007). This strategy resulted in the characterization of aspyridone and its biosynthetic genes in *A. nidulans*. Similarly, overexpression of a regulator residing next to two NRPS genes induced not only expression of these genes on chromosome II, but also led to induction of genes responsible for the synthesis of asperfuranone on chromosome VIII of *A. nidulans* (Bergmann et al. 2010). These results suggest that, in addition to metabolite-specific regulation, a regulatory cross-talk between different SM pathways also exists.

**Fig. 1:**
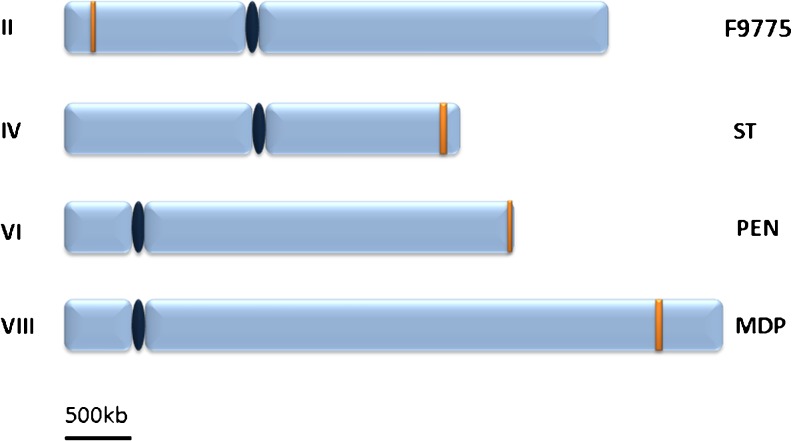
Subtelomeric localization of some well-studied SM gene clusters in *A. nidulans* marked in *yellow*. The orsellinic acid/F9775 cluster (ORS) is positioned on the left arm of chromosome II, ST—sterigmatocystin on the right arm of chromosome IV, PEN—penicillin cluster close to the end of chromosome VI's right arm, and MDP—monodictyphenon on the right arm of chromosome VIII

Genes involved in the biosynthesis of a certain metabolite are usually physically linked on the chromosome (clustered) and transcriptionally co-regulated. Although this clustering has already been observed before for some well-studied SM genes in *Aspergillus* and *Fusarium* species (Keller and Hohn 1997), large scale sequencing data revealed that clustering is the rule rather than the exception for SM biosynthetic pathways. Many examples are known for clustering of bacterial SM genes in large operons that transcribe poly-cistronic messages from a single or from multiple promoters (Malpartida and Hopwood 1984). The linkage of SM genes in fungi underpins the hypothesis that SM genes have been transferred from bacteria to ancestors of fungal lineages or between fungi. Horizontal gene transfer (HGT) events are well documented for β-lactam antibiotics (Brakhage et al. 2009) and have recently been found to be responsible for the presence of the sterigmatocystin gene cluster in *Podospora anserina* (Slot and Rokas 2011). Strikingly, the *A. nidulans* and *P. anserina* ST clusters are highly synthenic providing a strong argument for HGT of a complete cluster. Also, a certain bias for fungal SM cluster position near telomeres is an indication that these genes have been integrated into the fungal genomes as large units. It is known that subtelomeric regions and adjacent euchromatic DNA are highly recombigenic in many organisms and frequently contain repetitive elements (Barton et al. 2008; Linardopoulou et al. 2005). Tilburn et al. 1990 found repetitive elements in the region adjusted to *ωA* locus encoding for polyketide synthase involved in biosynthesis of the dark green pigment in the conidium wall. Also recently, repetitive DNA has been discovered flanking the *A. nidulans* PEN gene cluster which is located at the very end of the right arm of chromosome VI (Fig. 1). Deletion of the repetitive DNA reduced transcription of PEN genes and antibiotic production (Shaaban et al. 2010). Hence, it can be speculated that repetitive elements not only facilitated acquisition but also activation of large genomic segments coding for SMs. In addition, when a selectable marker is inserted into the region instead of the PEN cluster, this transgene is not correctly expressed. This was shown by Palmer and colleagues who found that repression of the marker gene is dependent on heterochromatin formation (Palmer et al. 2010; Palmer and Keller 2011). The authors proposed that repression is a consequence of telomere position effects. But as other SM clusters, which are not located near chromosome ends (see examples in Fig. 1), are also regulated by heterochromatin-dependent mechanisms (see below), it remains to be shown if sub-telomeric positioning exerts a considerable regulatory effect on other SM clusters as well.

Following the acquisition of these clusters by fungal ancestor lineages, cluster arrangement may have been maintained because the close proximity of the genes would allow a coordinated transcriptional control by chromatin-based mechanisms. We previously summarized the factors so far identified to play a role in heterochromatin formation and SM gene silencing in *A. nidulans* (Strauss and Reyes-Dominguez 2011). In this review, we update the picture of chromatin-based fungal SM cluster regulation and highlight similarities and differences between individual clusters within the same organism and between species.

## Regulation of gene activity by chromatin structure and histone modifications

**Fig. 2:**
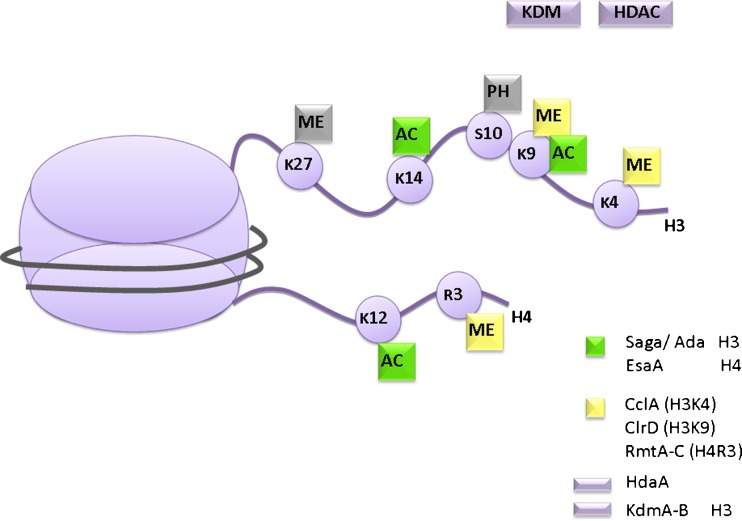
Known post-translational modifications of N-terminal tails in histones H3 and H4 of *A. nidulans. AC*—acetylation, *ME*—methylation and *PH*—phosphorylation have been shown to be present at several residues within histone proteins. The following regulators and enzyme complexes were determined to be involved in modification of residues: Saga/Ada multi-subunit complex containing GcnE as catalytic subunit for histone acetyltransferase activity; EsaA—H4 acetyltransferase putatively part of the NuA4 complex; CclA—a member of the putative Set1 histone H3K4 methyltransferase complex; ClrD—a H3K9 methyltransferase necessary for di- and trimethylation of H3K9; RmtA-C—histone H4R3 methyltransferases involved in oxidative stress response (Bauer et al. 2010); HDAC—histone deactylases, e.g., HdaA responsible for the main deacetylase activity in *A. nidulans*; KDM—histone demethylases: KdmA and KdmB are capable of removing the tri-methylation mark of H3K9 (Agnieszka Gacek and Joseph Strauss unpublished) but involvement of H3S10ph and H3K27me in controlling secondary metabolism is tentative

The natural substrate for the transcriptional machinery and regulatory proteins involved in chromosome segregation, DNA replication and repair in the eukaryotic nucleus is not DNA itself, but chromatin. It is composed of structural nuclear proteins such as histones and non-histone chromatin-associated proteins which are tightly associated with DNA and which condense the long DNA molecule into a small volume (Brown 1966; Kornberg 1974; Kornberg and Thomas 1974; Luger et al. 1997). Although packaging is essential and deletion of some chromatin-modifying factors is lethal, chromatin also represents a significant obstacle for DNA-binding factors to access their cognate sequences. Accessibility to chromatin is mainly regulated at the level of post-translational modifications (PTMs) of histone proteins (see examples in Fig. 2) which define the degree of compaction from loose (euchromatin) to very dense (heterochromatin). Protein modifications by acetylation, methylation, ubiquitination, and phosphorylation represent the main marks which are “written” onto different amino acids of N-terminal tails and globular domains of histone H2B, H3, and H4. During a regulatory cycle, these marks can be removed again by deacetylases, demethylases, and phosphatases, or one modification can be replaced by another on the same residue. This is the case in lysine modification, which can either be acetylated or methylated on the same nitrogen atom. Usually, the catalytic enzymes performing the specific addition or removal of the PTM are part of specialized histone-modifying complexes. Several of these complexes, such as the Saga/Ada or NuA4 acetyltransferase complexes, the SET-methyltransferase complexes or the histone deacetylase complexes are deeply studied, and for many members of these complexes, structural data as well as detailed mechanistic insights are available. Comparative analyses have shown that the basic mechanisms are conserved in eukaryotes (Aagaard et al. 1999; Allfrey et al. 1964; Allfrey and Mirsky 1964; Bannister et al. 2001; Bannister and Kouzarides 2011; Czermin et al. 2001; Jenuwein and Allis 2001; Richards and Elgin 2002; Vermeulen et al. 2010), with notable exceptions such as a loss of key components of heterochromatin in species of the *Saccharomycotina* sub-phylum (Hickman et al. 2011; Huang 2002; Rusche et al. 2003).

The dynamic activities carried out by “writing and erasing” modifications on histones result in a spatially and temporally changing combination of different histone marks at regulatory and coding regions of genes as well as in non-coding DNA stretches. These combinations are believed to generate a “code” that is recognized by “reader” proteins. Their role is to recruit transcriptional activators or repressors to these encoded locations (Bartke and Kouzarides 2011; Gardner et al. 2011; Kouzarides 2007; Li et al. 2007) and to change chromatin structure and compaction by interaction with factors responsible for the formation of heterochromatin. Because such histone marks, much like DNA methylation, can be stably transmitted during meiosis and over many mitotic cell cycles to daughter cells, the histone code has become an integrated component of epigenetic regulation. However, although epigenetic regulation employs histone marks, regulation at the chromatin level does not necessarily trigger an epigenetic event. In filamentous fungi, chromatin histone modifications and structure transitions have been studied mainly in the context of DNA methylation (Foss et al. 1993; Freitag et al. 2004; Kouzminova and Selker 2001; Tamaru and Selker 2001), light regulation (Grimaldi et al. 2006) in *Neurospora crassa*, in connection to the cell cycle (Osmani et al. 1991a; Osmani et al. 1991b) and nitrogen regulation (Berger et al. 2006; Berger et al. 2008; Reyes-Dominguez et al. 2008) in *A. nidulans*.

## Histone modifications and the chromatin landscape in SM gene clusters

**Fig. 3:**
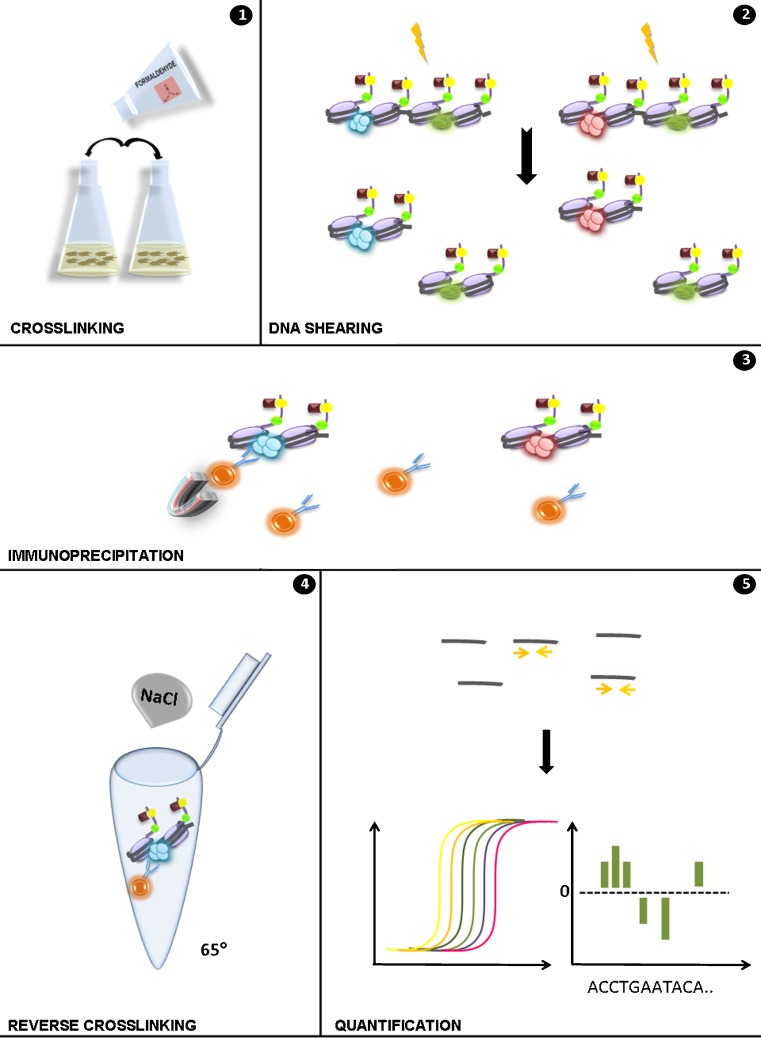
Technical outline of the Chromatin Immuoprecipitation (ChIP) procedure. (*1*) DNA and interacting proteins are cross-linked with formaldehyde. (*2*) Chromatin is shared into fragments of approximately equal size and precipitated with an antibody conjugated to magnetic beads and specifically recognizing the modification in question (*3*). After several washing steps, the precipitated complex of antibody—protein—DNA is reverse cross-linked by incubation at high temperature with NaCl (*4*). Purified DNA is quantified by qPCR using specific primers or analysed by massive parallel sequencing (MPS) (*5*)

The influence of histone modifying enzymes on SM production was first reported for ST production and gene regulation by Shwab et al. (2007). In this initial work, the authors found that *Aspergillus nidulans* HDACs mutants, which were previously generated and characterized in the laboratory of Steve Graessle, bypass the requirement for the general SM activator LaeA (Bok and Keller 2004). Because histone acetylation generally acompanies gene activation it was reassuring to observe that lack of deacetylases or inactivation of their activity by deacetylase inhibitors leads to higher SM production in several fungi including *Aspergillus*, *Penicillium*, and *Alternaria* species. Employing chromatin immunoprecipitation (ChIP, see Fig. 3 for a technical overview) in the sterigmatocystin cluster of *A. nidulans*, our group subsequently showed that LaeA only indirectly influences the acetylation status of histone H3 (Reyes-Dominguez et al. 2010). This influence is based on competitiveness of “labeling” the crucial lysine (K9) in histone H3, which is acetylated together with H3K14 during gene activation, but which is trimethylated at K9 in the repressed state. Trimethylation of H3 at K9 in turn provides a docking site for the “reader” heterochromatin protein 1 (HepA in *A. nidulans*, *hpo* in *N. crassa*) and this binding event leads to the formation of heterochromatic structures at this locus. Interestingly, deletion of HepA or the H3K9 methyltransferase ClrD leads to permanently open chromatin at the ST locus and, similar to deacetylase inactivation, to partial remediation of ST gene expression in the absence of LaeA. Strikingly, loss of LaeA function showed strongly elevated H3K9 methylation levels suggesting that LaeA counteracts H3K9 trimethylation and thus heterochromatin formation at the ST locus.

Reduction of H3K9me3 and HepA binding was also observed inside the newly identified monodictyphenon (MDP) cluster of *A. nidulans* as a consequence of the *cclA*Δ mutation (Bok et al. 2009). CclA is homologous to Bre2 in *Saccharomyces cerevisiae*, a protein that is part of the so-called COMPASS complex. This complex contains the SET1 methyltransferase that di- and trimethylates lysine 4 in histone H3 (H3K4me2/3). In a number of studies, this specific PTM was found to be associated with gene activation at euchromatic regions (Bernstein et al. 2005; Sims et al. 2007), but it has also been found as a mark required for subtelomeric gene silencing (Briggs et al. 2001; Mueller et al. 2006; Roguev et al. 2001). Most probably, in *A. nidulans*, the reduction of H3K9me3 at the MDP cluster in the *cclA*Δ strain is the consequence of a cross-talk between these two different PTMs of lysines in H3. But because the histone code is far more complex than has been tested here, we cannot say whether reduction of H3K4 methylation directly reduces H3K9 methylation (e.g., by blocking access of the ClrD H3K9 methyltransferase) or if this effect is mediated by some other, not tested, PTM at this locus.

Whatever the molecular mechanism may be, it is remarkable that H3K9me3 reduction and thus the removal of heterochromatic marks is specific for genes lying inside the MDP cluster, whereas genes not belonging to the cluster show reduced H3K4 methylation but high H3K9me3 heterochromatic marks. Cluster-specific PTMs have also been observed for the ST pathway. Reduction of H3K9me3 during the switch from primary to secondary metabolism was only seen in genes inside the ST cluster whereas genes lying immediately outside this biosynthetic region did not exhibit changes in their H3K9 methylation marks. How the borders between the heterochromatic and euchromatic domains are established and maintained still await clarification.

## SM cluster regulation by facultative heterochromatin

The two reports mentioned above (Bok et al. 2009; Reyes-Dominguez et al. 2010) were the first formal demonstrations that repression of SM gene expression during the active growth phase and primary metabolism employs the heterochromatin regulator HepA (HP1-homologue) bound to trimethylated histone H3K9. Because repressive heterochromatin marks were dynamic and dropped after initiation of secondary metabolism and ST gene transcription the ST region was considered “facultatively heterochromatic”. In contrast to constitutive heterochromatin which is formed at centromeric and telomeric regions and which does not undergo de-condensation, facultative heterochromatin is reversible and can switch to euchromatic structures. Although *bona fide* constitutive heterochromatin has not been studied in Aspergilli, there is a solid body of evidence for the existence of such structures in fungi such as *Schizosaccharomyces pombe* (Ekwall et al. 1995; Lejeune et al. 2010), and *Neurospora crassa* (Honda and Selker 2008; Honda et al. 2010; Honda et al. 2012; Lewis et al. 2010; Smith et al. 2008). However, during the heterochromatin–euchromatin switch silencing marks are replaced by activating marks, and this leads to the activation of genes in a specified genomic region (Brown 1966; Grewal and Jia 2007; Heitz 1928; Huisinga et al. 2006). Transcriptional silencing of genes by facultative heterochromatin formation is thought to be more tight than simple transcriptional repression in euchromatin because dense chromatin packaging represents a more restrictive and permanent barrier to the transcriptional machinery than repressor binding (Sun et al. 2001). Moreover, using chromatin condensation mechanisms, repressive structures can be more easily established over larger genomic regions. Because of these features, regulation by facultative heterochromatin is frequently found for developmentally regulated genes in higher eukaryotes (Fire et al. 1998; Kennerdell and Carthew 1998; Paddison and Hannon 2002). If developmental genes in fungi are associated with these chromatin structures is not known although mutations in the catalytic or regulatory subunits of the *A. nidulans* Saga/Ada complex (GcnE or AdaB) lead to a severe reduction of conidiation (Reyes-Dominguez et al. 2008) suggesting that histone marks and chromatin structure plays a central role at least in the asexual development of this fungus.

As a consequence of chromatin-level regulation, signaling pathways for SM activation must communicate with the chromatin modification machinery and implement this further up in the hierarchy level of regulation. The effects of HDAC inactivation and the detection of heterochromatic structures in the ST and MDP clusters provide an attractive hypothesis to explain co-regulation of large genomic regions and transcriptional silencing of SM biosynthetic genes during primary metabolism. In this model, secondary metabolic conditions would trigger the reversal of heterochromatic marks and the deposition of activating histone marks such as lysine acetylation in H3 and H4. In other words, both enzymatic activities removing repressive heterochromatic marks and placing activating euchromatic marks must be recruited to SM gene promoters under secondary metabolic conditions. Usually, HAT complexes are recruited to nucleosome-free regions in promoters of genes by specific DNA-binding proteins. This leads to a sequence of downstream events such as histone hyperacetylation, nucleosome remodeling, assembly of the basic transcriptional machinery, and subsequent RNA polymerase II recruitment culminating in transcriptional activity of the promoter (Berger 2002; 2007).

## The chromatin code is different for different SM clusters

Not for all studied SM clusters, a pathway activator performing the first step in recruitment is known and the broad-domain activator LaeA is not predicted to possess DNA-binding activity. For example, for the *A. nidulans*, penicillin or orsellinic acid/F9775 (ORS) (encoding for archetypical polyketide orsellinic acid and F9775A, F9775B cathepsin K inhibitors) clusters no pathway-specific activators have been identified and activation is mediated by general transcription factors (Espeso et al. 1993; MacCabe et al. 1990; Sanchez et al. 2009; Schroeckh et al. 2009). Still, the genes are strongly induced by SM-activating conditions. Are different activators responsible for recruitment of putative H3K9me3 lysine demethylases (KDMs) to reverse heterochromatic marks and for attracting HATs to place activating marks? At the moment, there is only scattered information available from chromatin studies at SM clusters and to answer these crucial questions is not yet possible. Moreover, only the apparently most relevant chromatin modifications have been studied so far but a much larger array of additional modifications are possible and may play an essential role in SM gene silencing and activation (Kouzarides 2007).

## Histone acetylation—an essential general mark for SM gene activation

In all eukaryotes, histones are acetylated at several positions during gene activation and several multi-subunit complexes such as Saga/Ada or NuA4 are known to possess histone acetyltransferase activities (Brownell and Allis 1995; Brownell et al. 1996; Grant et al. 1997; Grant et al. 1998). Whereas gene regulation by HATs was extensively studied in many organisms, comparatively little molecular information on the function of these complexes is available in filamentous fungi. In *N. crassa*, Grimaldi and collegues showed that the light-response regulator WC1 recruits the catalytic subunit NGF-1 to light regulated genes and catalyses H3K14 acetylation during gene activation (Grimaldi et al. 2006). In *A. nidulans*, the catalytic and regulatory subunits of Saga/Ada, GcnE, and AdaB, respectively, are required for induction of conidiation ((Reyes-Dominguez et al. 2008) and Ana Marcos, Agnieszka Gacek, Joseph Strauss, and David Canovas, unpublished results) and for regulation of nitrogen assimilation (Berger et al. 2008; Reyes-Dominguez et al. 2008). The strong stimulatory effect of HDAC-inhibitors on SM production in several fungi (see above) suggested a significant involvement of this histone modification in SM cluster regulation. Chromatin immunoprecipitation at the ST locus of *A. nidulans* using antibodies specific for acetylated H3K9 and H3K14 provided the first direct evidence that histone acetylation is increased under secondary metabolite production conditions (Reyes-Dominguez et al. 2010). That indeed the catalytic and regulatory subunits GcnE and AdaB are responsible for this increase in H3 acetylation was subsequently confirmed (Nützmann et al. 2011). Our work also demonstrated that, apart from ST, other genes belonging to the PEN, TR, and ORS clusters are also targets of the Saga/Ada co-activator complex. Consistently, the ORS cluster, which is not transcribed under standard secondary metabolite conditions, is strongly induced by addition of the HDAC inhibitor suberoylanilide hydroxamic acid (SAHA) and induction is blocked by addition of the HAT inhibitor anacardic acid (Nützmann et al. 2011). Remarkably, severe nitrogen limitation during growth in a fully a controlled chemostat, induced polyketide biosynthesis genes that were otherwise silent and yielded two novel antiproliferative spiroanthrones, sanghaspirodins A and B (Scherlach et al. 2011). This would suggest that the transcriptional co-activator AreA which senses the nitrogen status of the cell and conveys this information to nitrogen metabolite repressed genes (Berger et al. 2008; Caddick et al. 1986; Scazzocchio 2000; Schinko et al. 2010), is involved in ORS cluster activation. AreA dependence has not been directly tested for any *A. nidulans* SM cluster, however, a direct link between nitrogen regulation and Saga/Ada function has been demonstrated for primary metabolism in the nitrate assimilation pathway. In this model system, the GATA-factor AreA responds to intracellular glutamine (Gln) concentrations and is active under nitrogen limiting, low Gln conditions. Consequently, N-starvation or any other condition resulting in low Gln leads to histone H3 acetylation and chromatin remodeling in a strictly AreA-dependent manner (Berger et al. 2006; Berger et al. 2008; Muro-Pastor et al. 1999; Schinko et al. 2010). Interestingly, the production of sterigmatocystin is stimulated by NO_3_^−^ and repressed by NH_4_^+^ in *A. nidulans* cells, and microarray data of the nitrogen response confirmed the up-regulation of genes involved in the biosynthesis of SM precursors and ST genes under NO_3_^−^ conditions (Schinko et al. 2010). Unfortunately, a systematic study of AreA function in *A. nidulans* secondary metabolism is difficult because growth on nitrate, which is necessary for ST production, is not possible in *areA* loss-of-function strains.

AreA function in nitrogen regulation is conserved in all fungi and media shift experiments were performed to circumvent this problem in the plant pathogen *Gibberella fujikuroi.* In this organism, Giberellin (GA) production and the expression of the corresponding biosynthetic genes is under the control of FfAreA (Wagner et al. 2010). Whether AreA is acting only as transcriptional co-activator or also required for histone acetylation and subsequent chromatin remodeling at the GA cluster awaits clarification.

**Fig. 4:**
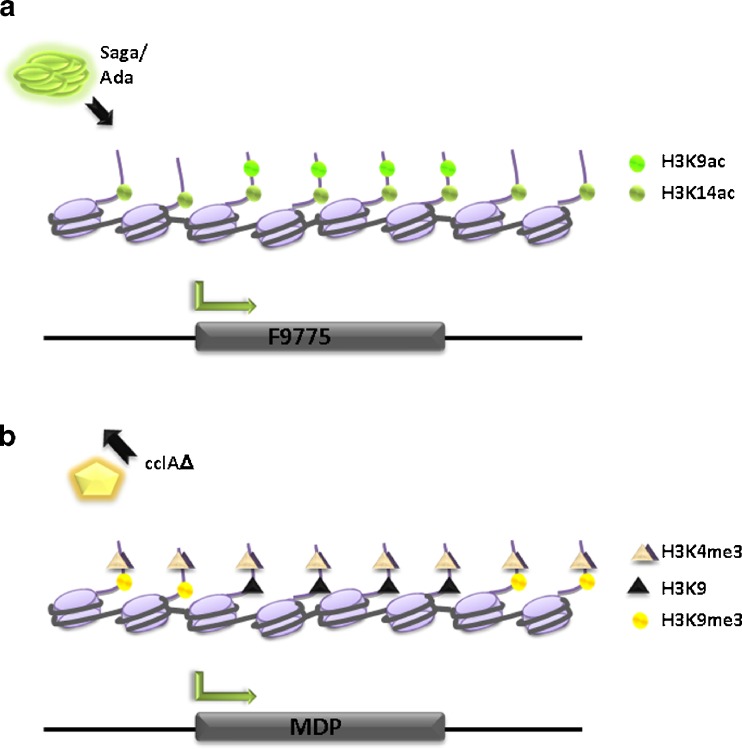
Activation of SM gene clusters. In some cases the recruitment of positive regulators is required to activate silent SM gene clusters, whereas in other cases removal of negative regulators is sufficient. **a** Co-incubation of *A. nidulans* with *S. rapamycinicus* leads to increased acetylation of histone H3 lysine 9 (cluster specific) and lysine 14 (cluster unspecific) what results in subsequent activation of the ORS cluster. **b** Deletion of CclA leads to reduced H3K4me3 levels at a large genomic region including the MDP gene cluster however only at genes belong to the cluster H3K9me3 is decreased, what is sufficient to activate the otherwise silent MDP pathway

Increasing the level of histone acetylation at SM clusters was also found to be the molecular basis of how bacterial-fungal co-cultivation activates silent clusters in *A. nidulans*. In an earlier study, Schroeckh and coworkers (2009) reported that direct physical interaction of *A. nidulans* and a specific strain of the soil-dwelling bacterium *Streptomyces rapamycinicus* resulted in the activation of a large silent cluster containing biosynthetic genes for orsellinic acid, lecanoric acid, and the cathepsin K inhibitors F-9775A and F-9775B. In a recent follow-up study, Nützmann and colleagues (2011) could demonstrate that bacterial co-cultivation generates the signal for Saga/Ada-mediated H3K9 and K14 acetylation, providing a molecular explanation for the ORS cluster activation under these conditions. Because inhibition of histone deacetylases by SAHA phenocopies bacterial co-cultivation bacteria could generate a signal that hyper-activates Saga/Ada or inhibits H3 deacetylation, or both. The fact that we found by ChIP more GcnE-FLAG at ORS cluster promoters during co-cultivation, however, indicates that increased Saga/Ada activity is at least one mechanism contributing to the observed effect. It is interesting to note that H3 acetylation marks are different inside and immediately outside the ORS cluster (Fig. 4a). Whereas H3K14 acetylation can be found inside the cluster but also in the neighboring region, H3K9 hyperacetylation following bacterial stimulation can only be detected within the cluster. The crucial role for H3K9 in defining the borders of a cluster was already shown for ST (Reyes-Dominguez et al. 2010) and for MDP (Bok et al. 2009) (see Fig. 4b). Therefore, this mark surely plays a key role in defining the transcriptional activity of genes belonging to a defined cluster and might even be used to predict the limits of unknown biosynthetic clusters. If the respective H3K9 modification is involved in setting the exact borders or if the pre-defined borders define H3K9 marks between these limits is currently unknown.

**Fig. 5:**
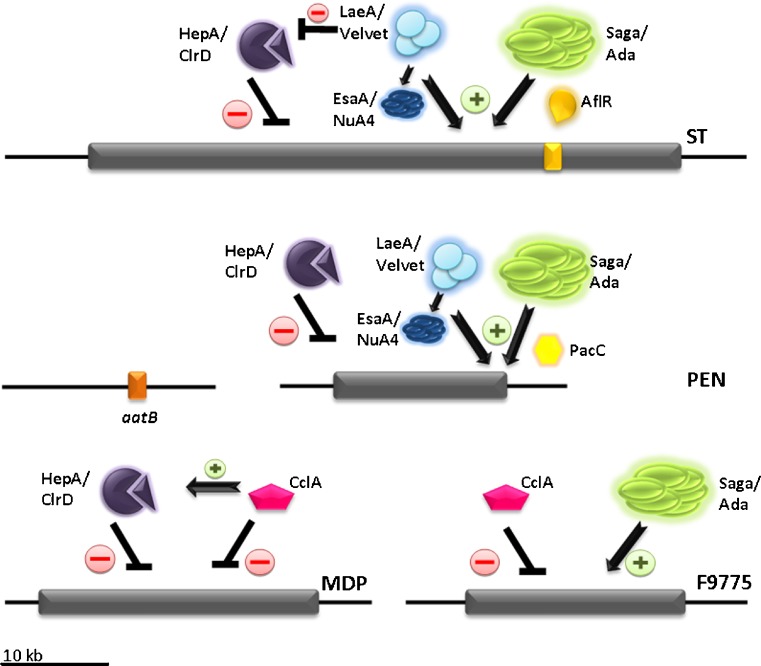
Different SM biosynthetic clusters respond to different activation pathways. Some of the clusters possess pathway specific activators, e.g., AflR regulating the ST cluster, while in some clusters the involvement of broad domain regulators have been shown, e.g., the pH regulator PacC is required to turn on the PEN cluster. Functional and probably physical interaction between HepA and ClrD negatively regulates the expression of all tested SM gene clusters. The function of CclA, part of the putative Set1 methyltransferase complex, was found to only inhibit the expression of genes within the MDP and ORS clusters, partially via a positive action on HepA/ClrD. The complexity of this regulatory network is illustrated by the interplay between LaeA, which negatively influence HepA/ClrD function but stimulates the function of EsaA in acetylating H4K12. Interestingly, the *aatB* gene involved in penicillin biosynthesis but located on chromosome I outside of PEN cluster, is not co-regulated by chromatin remodelling proteins. *Arrows* and *plus* symbols indicate activation, block lines, and minus symbols represent inhibition

How the signal for Saga/Ada activation or HDAC inactivation is generated also remains a matter of speculation, especially as an intimate contact between the organisms is required to trigger this effect. One possibility might be nutrient competition, but the fact that only one specific isolate of many strains tested was able to trigger the activation event makes this mechanism unlikely. Another or an additional trigger could be some sort of stress imposed on *A. nidulans* cultures by bacterial cells. In support of such a mechanism Linz and colleagues recently found that the oxidative stress pathway mediated by cAMP-response elements is involved in the activation of the aflatoxin gene cluster in *A. parasiticus* (Roze et al. 2011). Thus, one of several plausible signaling pathways to unlock orsellinic acid production by bacterial contact could also involve an oxidative stress-related pathway that co-activates Saga/Ada. Although involvement of Saga/Ada or H3 acetylation has not been reported for SM regulation in *A. parasiticus*, it is known that histone H4 hyper-acetylation parallels the activation of genes within the aflatoxin cluster (Roze et al. 2007). H4 acetylation also seems to play a role in *A. nidulans* SM regulation. Four gene clusters were recently examined for this chromatin mark (sterigmatocystin, penicillin, terrequinone, and orsellinic acid) by Soukup et al. (2012). In this work, the authors found augmented H4K12 acetylation in the tested SM clusters during the secondary metabolite production phase. Based on suppressor screens and overexpression studies, the essential EsaA histone acetyltransferase was suggested to be responsible for this chromatin acetylation pattern. EsaA is the *A. nidulans* orthologue of Esa1, the catalytic subunit of the essential *S. cerevisiae* NuA4 transcriptional adaptor/acetyltransferase complex involved in H2A and H4 acetylation, cell cycle control, and epigenetic control of transcription (Allard et al. 1999; Doyon and Cote 2004; Galarneau et al. 2000). Clearly, H4 acetylation by EsaA requires functional LaeA, suggesting that this general activator of SM and member of the velvet complex (Bayram and Braus 2011; Bayram et al. 2008; Sarikaya Bayram et al. 2010) participate in recruitment of the putative *A. nidulans* NuA4 complex. This is in contrast to H3 acetylation where this general regulator does not seem to play an essential role in recruitment of the Saga/Ada complex. (Figure 5 shows a side-by-side comparison of the best-studied clusters). This view is supported by the finding that bacteria-induced transcription of the ORS cluster is LaeA-independent (Nützmann et al. 2011) and that Saga/Ada-mediated H3 acetylation of the tested ST-cluster genes is largely independent of LaeA (Reyes-Dominguez et al. 2010). In conclusion, both Saga/Ada-mediated H3 and NuA4-mediated H4 acetylation appear to be essential for induction of several SM gene clusters but only H4 acetylation seems to directly rely on functional LaeA. H3 acetylation is influenced by LaeA function indirectly by defining the methylation status of H3K9.

## LaeA and the crosstalk to histone methylation

In contrast to acetylation, which basically always reads out as an activating chromatin mark, histone methylation represents a much more complex language. Methylation of H3K9, H3K27, and H4K20 are most frequently associated with heterochromatin and transcriptional silence whereas H3K4, H3K36, and H3K79 methylation are most often found—together with hyperacetylation of H3K9—in actively transcribed euchromatin. Acetylation and methylation are exclusive on lysines and can never co-exist at the same time on the same residue. But this is only part of the truth: depending on the degree of methylation (mon-, di-, or trimethylation) of a given lysine, whether the mark is posted on a histone residing in a promoter region or in the coding sequence, and which chromosomal location is affected, any given methylation mark can either be read as an activating or repressing signal (Brosch et al. 2008; Cichewicz 2009; He and Lehming 2003; Kouzarides 2002; Zhang and Reinberg 2001) .

A small window of this enormous complexity has been opened by the finding that *A. nidulans* LaeA greatly influences the methylation state of H3K9 and subsequent occupancy of this locus by HepA. Binding of HepA to chromatin requires di- and tri-methylation of H3K9 (Bannister et al. 2001; Reyes-Dominguez et al. 2010). Furthermore, in *S. pombe* it was shown that SWI6 (the HP1 homologue) interacts with and recruits the H3K9 methyltransferase Clr4 (Ekwall et al. 1996). These tripartite interactions promote the establishment of extended heterochromatic domains by repeated binding and methylation cycles. Consistent with the situation described in *S. pombe*, a lack of HepA led to loss of H3K9me3 in the ST cluster of *A. nidulans*, suggesting that HepA recruits ClrD, the orthologue of Clr4 in *A. nidulans.* The fact that loss of LaeA function results in strongly elevated H3K9me3 and HepA levels in the ST cluster and that HepA deletions partially bypass the requirement for LaeA for transcription of several SM clusters, advocated a role of this regulator in heterochromatin formation and transcriptional repression. The precise mechanism of LaeA function in this process remains unidentified but we already proposed that the protein could be directly or indirectly involved in blocking heterochromatin formation. ClrD and HepA function could be affected and the function of histone H3K9 demethylases could be stimulated by LaeA. The fact that HepA deletion is only partially remediating a *laeA* mutant phenotype for ST gene transcription and metabolite production (Reyes-Dominguez et al. 2010) suggests that LaeA and HepA function to some extent in the same pathway but LaeA function must reach beyond counteracting HepA. Two putative H3K9 demethylases are present in the *A. nidulans* genome (our unpublished observation) and one or both proteins could cooperate with LaeA to demethylate H3K9me3 within SM clusters under the specific metabolic conditions. Preliminary evidence from our laboratory suggests that both *A. nidulan*s KDMs are functional and play a role in SM regulation (Agnieszka Gacek and Joseph Strauss, unpublished observations).

## Does heterochromatin also regulate SM in other fungi?

**Fig. 6:**
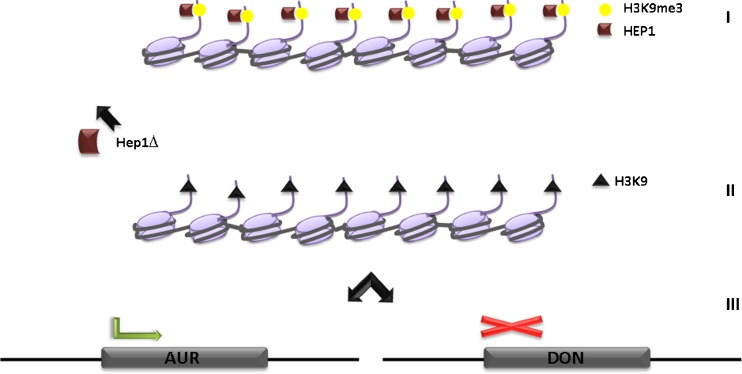
Regulation of SM gene expression by chromatin structure in *F. graminearum*. *I*. During primary metabolism, deoxynivalenol (DON) and the aurofusarin (AUR) clusters are silent and marked by HEP1 (ortholog of *A. nidulans* HepA) bound to H3K9me3. *II.* Deletion of Hep1 leads to decreased H3K9me3 levels (equal to all tested SM clusters in *A. nidulans*) on promoters of DON and AUR biosynthetic genes. *III.* Absence of heterochromatic marks results, on one hand, in the activation of aurofusarin and repression of deoxynovalenol biosynthetic genes

Beside from what has been described above for *A. nidulans* and *A. parasiticus*, chromatin in relation to SM gene expression has been studied only in a few other organisms. It should be noted here that heterochromatin formation in filamentous fungi is without doubt best understood in *N. crassa* (Rountree and Selker 2010), but this organism is not a suitable model for secondary metabolism. In contrast to LaeA, which is well studied at the genetic, genomic, and physiological level and known to regulate chemical diversity in a large range of fungi (Bok and Keller 2004; Kale et al. 2007; Kale et al. 2008; Kosalkova et al. 2009; Oda et al. 2011; Perrin et al. 2007; Wiemann et al. 2010; Xing et al. 2010), factors regulating heterochromatin formation in SM gene clusters have only been studied in the human opportunistic pathogen *Aspergillus fumigatus* and the plant pathogen *Fusarium graminearum*. Deletion of the *A. fumigatus* H3K9 methyltransferase ClrD reduced viability and conidia production but did not change host interactions in a macrophage assay (Palmer et al. 2008). The effect of *clrD* deletion on chromatin structure, histone marks, and secondary metabolism has not been reported yet for *A. fumigatus*,

In contrast, a clear effect on secondary metabolism is seen in the heterochromatin protein 1 (*hep1*) deletion strains of the plant pathogen *F. graminaerum* (Reyes-Dominguez et al. 2012). Similar to *A. nidulans hepA* deletion strains, H3K9me3 is strongly reduced in the *F. graminaerum hep1* null mutants in two examined gene clusters (deoxynivalenol-DON) and aurofusarin AUR pigment). As expected, the absence of HEP1 and H3K9me3 leads to the activation of the aurofusarin gene cluster and consequently strongly elevated metabolite production. Surprisingly, the same mutation leads to gene repression of two genes belonging to the DON cluster and concomitant absence of the corresponding metabolite in liquid cultures (overview presented in Fig. 6). The interpretation of these results remains speculative but one possibility would be chromatin-based up-regulation of the repressor for DON production (Kimura et al. 2003; Kimura et al. 2007) in the *hep1* mutant.

In any case, the finding that *hep1* deletion strongly affects secondary metabolism in *F. graminearum* supports a more general role for facultative heterochromatin in fungal secondary metabolism. Also for this fungus sequencing information predicts a vastly higher number of biosynthetic clusters than the number of known metabolites. Efforts to de-repress these clusters of unknown function by modulating culture conditions have been only partially successful (Cuomo et al. 2007; Li et al. 2012; Seong et al. 2009) and mutants involved in heterochromatin formation might be helpful for the identification of novel metabolites from this agronomically important fungus.

## Concluding remarks and perspectives: similarities, differences, and current limitations

Although information on chromatin-level regulation of fungal secondary metabolite gene clusters is still scattered, some general conclusions from the published work can be drawn with confidence. From the actual evidence, it is safe to presume that histone H3 acetylation in promoter regions of SM cluster genes by GcnE-containing complexes is essential for transcriptional activation of SM clusters in *A. nidulans*. In addition, histone H4 acetylation has been shown to convoy transcriptional activation of several clusters in *A. nidulans* and *A. parasiticus* and thus seems to play a role during the activation process. However, the putative H4 acetyltransferase *esaA* is essential in *A. nidulans* and thus the quantitative contribution of this protein to SM gene activation is difficult to assess. But based on over-expression studies, which demonstrated a roughly two to three-fold higher transcript abundance of SM cluster genes, we can assume that the contribution of H4K12ac is probably less important than H3 acetylation.

The main difference between H3 and H4 acetylation is their dependence on LaeA: whereas H4 acetylation, at least in *A. nidulans,* is strictly dependent on LaeA, GcnE-mediated H3 acetylation seems to be largely independent of this general regulator. This means that GcnE-containing complexes (such as the putative Saga/Ada complex) are recruited to SM gene promoters by an alternative mechanism to acetylate at least H3K9 and K14 (these two modifications were tested with H3 K9 and K14 acetyl-lysine specific antibodies). Based on the available evidence the GATA-factor AreA, known to recruit acetylation activities to the nitrate promoters, might be a suitable candidate for GcnE/Saga recruitment to ST and other nitrogen-responsive biosynthesis pathways. For the pH-regulated PEN cluster, the general regulator PacC might take over this recruitment role.

LaeA not only acts as general SM activator, but to some extent also functions to detain specific chromatin modifications within the borders of active SM clusters. In recent studies high levels of H3acetylation at K9 and K14 were found to be confined to regions inside the transcriptionally active SM clusters. Conversely, adjacent genes not belonging to the clusters displayed only elevated H3K14 acetylation under these conditions (Nützmann et al. 2011). On the other hand, H3K9 was found to be highly trimethylated in primary metabolism and reduction of this mark during SM gene expression occurred again only within clusters and in *laeA* ^+^ strains, but not in genes lying immediately outside the cluster or in *laeA* mutants (Bok et al. 2009; Reyes-Dominguez et al. 2010). Thus, competing types of H3K9 modifications seem to carry out crucial regulatory functions for SM cluster expression. Based on this modification pattern, we can predict that LaeA—in concert with the *velvet* complex and probably other proteins—is part of a cluster-specific regulatory complex that keeps H3K9 methylation and subsequent HP1 association low. However, apart from blocking H3K9 methylation or promoting de-methylation and subsequent reversal of heterochromatic structures, the LaeA-*velvet* complex must have an additional role in SM gene activation. This can be concluded because deletion of components required for heterochromatin formation, i.e., the *clrD* H3K9 methyltransferase and *hepA*, encoding heterochromatin protein 1, only partially restores SM production. The exact molecular mechanism behind this additional function of LaeA is still a matter of speculation and subject to intense research.

Further studies are needed to obtain a more detailed picture of the regulatory network that remodels the repressive heterochromatin to transcriptionally competent euchromatin during the secondary metabolism phase. The greatest challenge in deciphering the chromatin code of SM clusters and adjoin regions is the availability of suitable antibodies specific for the large diversity of histone modifications in the studied organism. Although the histone PTMs are conserved structures in all eukaryotes, we can not exclude *a priori* other modifications or new combinations of known modifications in fungi. In addition, the interaction of the antibody with its epitope during ChIP can potentially be influenced by adjacent PTMs in neighboring amino acids. Therefore, each antibody in use has to be tested for specificity and control strains carrying amino acid changes in the modified residue of histones need to be generated. Some of these changes may not be possible at all because the resulting strain may not be viable. Moreover, the mutation itself might interfere with primary and secondary metabolite gene expression and therefore complicate interpretation of results. However, the advent of extremely powerful massive parallel sequencing techniques will help to enlarge the picture from specific SM clusters to a genome-wide scale and will provide valuable insights how chromatin structure and histone modifications react to metabolic changes and to mutations or over-expression of crucial regulators. And as the family of sequenced fungal genomes constantly grows, these approaches have the potential to draw out exciting data for comparative chromatin studies.

From the few well-studied model pathways, such as ST, PEN or the ORS cluster, we have already learnt that all clusters are controlled by a chromatin-based mechanism, but that the exact mode of control can differ significantly between the individual clusters within one organism. Even more so, for a huge number of sequenced fungal gene clusters predicted to code for biosynthetic activities, no corresponding metabolite has been identified so far. A better understanding of the chromatin-based regulatory network will certainly contribute to the discovery of novel fungal metabolites.
